# Discovery of Novel Biomarkers by Microarray Analysis of Peripheral Blood Mononuclear Cell Gene Expression in Benzene-Exposed Workers

**DOI:** 10.1289/ehp.7635

**Published:** 2005-03-14

**Authors:** Matthew S. Forrest, Qing Lan, Alan E. Hubbard, Luoping Zhang, Roel Vermeulen, Xin Zhao, Guilan Li, Yen-Ying Wu, Min Shen, Songnian Yin, Stephen J. Chanock, Nathaniel Rothman, Martyn T. Smith

**Affiliations:** ^1^School of Public Health, University of California, Berkeley, California, USA;; ^2^Division of Cancer Epidemiology and Genetics, National Cancer Institute, Bethesda, Maryland, USA;; ^3^National Institute of Occupational Health and Poison Control, Chinese Center for Disease Control and Prevention, Beijing, China

**Keywords:** Affymetrix, benzene, biomarkers, blood, expression profiling, leukemia, lymphocyte, microarray, molecular epidemiology, occupational exposure, real-time PCR

## Abstract

Benzene is an industrial chemical and component of gasoline that is an established cause of leukemia. To better understand the risk benzene poses, we examined the effect of benzene exposure on peripheral blood mononuclear cell (PBMC) gene expression in a population of shoe-factory workers with well-characterized occupational exposures using microarrays and real-time polymerase chain reaction (PCR). PBMC RNA was stabilized in the field and analyzed using a comprehensive human array, the U133A/B Affymetrix GeneChip set. A matched analysis of six exposed–control pairs was performed. A combination of robust multiarray analysis and ordering of genes using paired *t*-statistics, along with bootstrapping to control for a 5% familywise error rate, was used to identify differentially expressed genes in a global analysis. This resulted in a set of 29 known genes being identified that were highly likely to be differentially expressed. We also repeated these analyses on a smaller subset of 508 cytokine probe sets and found that the expression of 19 known cytokine genes was significantly different between the exposed and the control subjects. Six genes were selected for confirmation by real-time PCR, and of these, *CXCL16*, *ZNF331*, *JUN*, and *PF4* were the most significantly affected by benzene exposure, a finding that was confirmed in a larger data set from 28 subjects. The altered expression was not caused by changes in the makeup of the PBMC fraction. Thus, microarray analysis along with real-time PCR confirmation reveals that altered expressions of *CXCL16*, *ZNF331*, *JUN*, and *PF4* are potential biomarkers of benzene exposure.

Benzene is an important industrial chemical (> 2 billion gallons produced annually in the United States) and component of gasoline (Gist and Burg 1997). Its toxic effects on the blood and bone marrow include leukopenia, pancytopenia, and aplastic anemia, and it is also an established cause of human leukemia (Snyder 2002). However, the mechanisms of benzene-induced hematotoxicity and leukemo-genesis remain unclear, as does the risk benzene poses at low levels of exposure (Krewski et al. 2000). To shed further light on these mechanisms and better understand the risk benzene poses, we examined the effects of benzene exposure on peripheral blood mononuclear cell (PBMC) gene expression in a population of shoe-factory workers with well-characterized occupational exposures to benzene using cDNA microarrays and real-time polymerase chain reaction (PCR).

Microarrays use immobilized cDNA or oligonucleotide probes to simultaneously monitor the expression of thousands of genes and obtain a view of global gene expression (i.e., a view of all mRNA transcripts expressed by a cell is known as the transcriptome) (Staudt 2003; Staudt and Brown 2000) and are becoming increasingly used in toxicology (Hamadeh et al. 2002; Waters et al. 2003). They have also been used recently to investigate variation in gene expression in the peripheral blood leukocytes of normal individuals (Whitney et al. 2003). We hypothesized that microarrays could identify changes in gene expression that could be used as new biomarkers of exposure and early effect for benzene and provide information on mechanisms of benzene toxicity.

One potential problem with using microarrays in epidemiologic studies is that mRNA is unstable (Thach et al. 2003). Most epidemiologic studies that have collected biologic samples have not collected material that contains stabilized RNA for analysis. Here, we have overcome this problem by performing the first step of RNA isolation in the field and stabilizing the RNA for later analysis. We have analyzed this RNA from selected subjects using a comprehensive and standardized human array, the U133A/B Affymetrix GeneChip set (Iacobuzio-Donahue et al. 2003). U133A and U133B chips together contain almost 45,000 probe sets, representing > 39,000 unique transcripts derived from approximately 33,000 well-substantiated human genes, allowing investigators to obtain a global view of gene expression.

We performed a proof-of-principle study in which we examined global gene expression in a small number of well-matched exposed–control subject pairs. Genes with differential expression were then ranked and selected for further examination using several forms of statistical analysis. We also specifically examined the expression of all cytokine genes on the array under the *a priori* hypothesis that these key genes involved in immune function are likely to be altered by benzene exposure (Aoyama 1986). We then attempted to confirm the array findings for the leading differentially expressed genes using real-time PCR, which is thought to be more accurate than microarray analysis but can be used only to investigate a few genes at a time (Etienne et al. 2004). Once these genes were confirmed in the paired analysis, we examined their expression in a larger number of benzene-exposed subjects and controls. The overall goal is to provide potential gene markers of exposure and early effect for benzene and to produce mechanistic insight into how benzene affects the body, especially the immune system and lymphocyte function.

## Materials and Methods

### Study subjects and exposure assessment.

We studied workers exposed to benzene in two shoe manufacturing factories and unexposed controls from three clothes manufacturing factories in the same region of Tianjin, China. The study was approved by institutional review boards at all institutions. Participation was voluntary, written informed consent was obtained, and the participation rate was approximately 95%.

An initial group of six workers was selected from among the more highly exposed workers (mean benzene ± SD = 47.3 ± 24.3 ppm), and six controls were frequency-matched to these subjects on the basis of age and sex. Mean age was 33.7 ± 7.1 years for the six exposed workers and 31 ± 6.7 years for the controls. Four pairs were male and the other two female.

Before phlebotomy, individual benzene and toluene exposure was monitored by each wearing an organic vapor passive monitor badge as previously described (Vermeulen et al. 2004). Personal full-shift air monitoring was conducted about every month over a 3- to 4-month period before biologic sample collection. Benzene and toluene were not detected in air samples from the control factories.

Each subject was given a physical exam by a study physician. A questionnaire was administered that requested detailed information on occupation, environmental exposures to solvents and pesticides, past and current tobacco and alcohol use, past and current medical history including recent infections, diagnostic and therapeutic ionizing radiation exposure, medication use, family history, and a food frequency questionnaire developed for use in northern China.

### Biologic sample collection.

Peripheral blood, buccal cells, and urine were collected from each subject at the beginning of the workday around 0900 hr and were processed within 6 hr of collection. White blood cell differential counts and the levels of natural killer (NK) cells, B lymphocytes, CD4^+^ and CD8^+^ T lymphocytes were determined. The PBMC fraction, consisting of lymphocytes, monocytes, and some platelets, was isolated in the field using Ficoll-Paque (Amersham, Piscataway, NJ). One to five million PBMCs were added to 1 mL RLT buffer (Qiagen, Valencia, CA) containing 1% β-mercaptoethanol to preserve RNA in the cells. RNA that is frozen in this buffer at −80°C is highly stable.

### RNA isolation, amplification, and hybridization.

We isolated total RNA using RNeasy mini kits (Qiagen) according to manufacturer instructions and quantified using a SmartSpec 3000 (Bio-Rad, Hercules, CA). Only samples with an A260/A280 between 1.7 and 2.2 were considered suitable for use. Samples were prepared according to the GeneChip Eukaryotic Small Sample Target Labeling Assay Version II (Affymetrix 2003a), with the exception that the GeneChip Sample Cleanup Module (Affymetrix, Santa Clara, CA) was used and not ethanol precipitation. Total RNA (100 ng) was amplified for each sample, with 400 ng of first-round cRNA used for the second round of cDNA synthesis. Second-round cRNA (15 μg) was fragmented in 30 μL of 1× fragmentation buffer. Hybridization cocktails were made as described in the *GeneChip Expression Analysis Technical Manual* (Affymetrix 2003b) and hybridized to U133A chips at 60 rpm, 45°C. After 16 hr, the hybridization cocktails were removed, added back to the unused hybridization cocktails, and stored at −80°C. GeneChips were stained with streptavidin–phycoerythrin using the EukGE-WS2 protocol (Affymetrix 2003b). GeneChips were scanned twice using a GeneChip Scanner GA 2500 (Affymetrix). Frozen hybridization cocktails were heated to 65°C for 5 min and then applied to U133B chips as described for U133A chips [of the 45,000 probe sets, only 100 (which can be used for normalization) are found on both chips, so this “recycling” of hybridization cocktail should not affect the results]. Chips were then analyzed as described below.

### Chip normalization.

To allow comparison, all chips were scaled to a target intensity of 500 based on all probe sets on each chip. Samples were run blind so that exposure status was unknown and designated as being either group A or B. Group A chips were used as the baselines when analyzing chips from group B.

### Statistical analysis to identify differentially expressed genes.

Robust multiarray analysis (RMA) (Irizarry et al. 2003) was used to analyze the data produced by the chips. Two RMA analyses of the GeneChip data were performed. First, we performed a global gene analysis that looked at all genes on both chips simultaneously. Probe sets for which expression was significantly different between exposed and unexposed individuals were identified using a standard paired *t*-test, and a recently developed bootstrapping technique to provide a critical value adjusted to provide a 5% familywise error rate (FWER), the standard value used in the literature. The bootstrapping technique can provide a more accurate (and less conservative) FWER than standard methods (e.g., Bonferroni’s adjustment) (Pollard and Van der Laan 2003). As Dudoit et al. (2004) noted, bootstrapping resampling techniques can be used to directly model the joint distribution of the null test statistics so that the dependence of genes is implicitly factored in when determining error rates for different cutoffs. The main advantage of this technique over others such as Bonferroni’s is that it can provide accurate control of error rates even when gene expressions on the same chip are statistically dependent (in this case, Bonferroni is often very conservative). However, the theory developed for the technique is asymptotic, and its performance can be less than optimal with very small sample sizes (due to random sampling of matched array pairs with replacement causing an excessive number of ties in some samples).

RMA (normalization, background correction, and calculation of expression) was applied to all genes and all chips simultaneously. We then performed a targeted analysis of cytokine genes by applying the multiple testing procedures to this subset after RMA processing was completed. This was based on the *a priori* hypothesis that cytokines involved in the immune response should be affected by benzene exposure because of its known immunotoxicity, and we thus derived more power to select differentially expressed cytokine genes by limiting the analysis only to this subset. The subset of 508 cytokine probe sets represented on the U133 chips was identified using NetAffx (http://www.affymetrix.com/analysis/index.affx) and the key word “cytokine.”

### Real-time PCR confirmation using TaqMan.

Total RNA (100 ng) was converted to cDNA using the SuperScript First-Strand Synthesis System for reverse-transcriptase PCR (Invitrogen, Carlsbad, CA) using oligo dT primers according to the manufacturer’s instructions. This cDNA was used to confirm GeneChip findings using TaqMan Gene Expression Assays (TMGEAs; Applied Biosystems, Foster City, CA). Assays were run in quadruplicate with 1× TaqMan Master Mix (Applied Biosystems), 1× assay mix, and 50 ng of cDNA in each 25-μL reaction for six genes plus TATA box binding protein (*TBP*) as an endogenous control (32 reactions/sample). Reactions were run on Applied Biosystems ABI PRISM 7700 Sequence Detection System as follows: 95°C for 10 min, followed by 40 cycles of 95°C for 15 sec, 60°C for 1 min. The 12 samples that had been run on chips were run in exposed–unexposed pairs to reduce experimental variability. The mean baseline (*TBP*) threshold cycle (*C*_t_) was subtracted from the mean *C*_t_ for the other six assays to normalize results. These were then compared between exposed and unexposed sample pairs. Assays used were as follows: *TBP* (endogenous control), Hs99999910_m1; *CXCL16*, Hs00222859_m1; *IL4R*, Hs00166237_m1; *JUN*, Hs00277190_s1; *PF4*, Hs00427220_g1; *PTPRE*, Hs00369944_m1; *ZNF331*, Hs00367929_m1.

## Results

### Differential global gene expression in the exposed–control matched pairs.

PBMC RNA from six matched pairs of subjects (one exposed and one age- and sex-matched control in each pair) was analyzed on Affymetrix GeneChips. RMA analysis of the data using paired *t*-statistics, bootstrapping, and a 5% FWER indicated that 2,129 probe sets were significantly different in people exposed to high levels of benzene compared with matched unexposed subjects. Expression of 964 of these probe sets was decreased, and 1,165 were increased. Table 1 shows the top 25 up-regulated probe sets identified on the basis of the lowest *p*-values, and Table 2 shows the top 25 down-regulated probe sets. A number of the probe sets identified in the top 50 were expressed sequence tags (ESTs) or coded only for hypothetical proteins. Twenty-nine probe sets corresponded to genes coding for known proteins. Of these, the gene for *HSPA1A* was the most strongly down-regulated (−66%) (Table 1), and that for *CREM* was the most strongly up-regulated (+145%) (Table 2). The significance of this latter finding is unclear because *CREM* has very low expression in lymphocytes. Other genes of note that were up-regulated were *ZNF331*, *PTPRE*, toll-like receptor 2, the chemokine *CXCL16*, and *CD44* antigen (Table 1). Note that *CD44* and *ZNF331* are present on both A and B chips and so they are listed twice in Table 1, but both have similar *p*-values and expression ratios on the A and B chips, providing a good quality control check. Other genes of interest that were significantly down-regulated include the oncogene *JUN*, *MAP4*, and *CALM1* (Table 2).

### Differential cytokine gene expression in the exposed–control matched pairs.

RMA analysis of the subgroup of 508 cytokine probe sets on the chip indicated that the expression of 19 cytokine genes was significantly different between the exposed and control subjects (Table 3). *IFNGR1*, *IL6R*, *CCNT2*, *PBEF1*, and *PPP1CB* were identified by two probe sets, so 28 identification numbers (IDs) are listed in Table 3. The 19 differentially expressed cytokine genes were also identified in the global analysis, but only a few had *p*-values low enough to be listed in Tables 1 and 2. However, several had high ratios of differential expression between exposed and controls, with *PBEF1*, *IFNGR1*, and *CXCL16* being increased around 100% (Table 3). Interestingly, six of the up-regulated genes were receptors for interleukins 2, 4, 6, 10, and 11 and interferon gamma, the latter being the most strongly down-regulated cytokine gene. *PF4* was the second most significantly down-regulated gene (Table 3).

### Confirmation by real-time PCR.

Four genes—*CXCL16*, *JUN*, *PTPRE*, and *ZNF331*—were chosen from the global analysis and two genes—*L4R*, *PF4*—from the cytokine subset for further study and confirmation by real-time PCR. We selected the global analysis genes for further study by first removing ESTs, hypothetical proteins, and genes with low levels of expression. From the remaining genes, we used magnitude and direction of change and availability of TMGEAs at the time of this analysis to decide which to confirm by real-time PCR. Using these three parameters, we chose three of the most significantly up-regulated genes and one strongly down-regulated gene (*JUN*) for confirmation. *IL4R* and *PF4* were chosen for confirmation from the cytokine subset because they were, respectively, the most significantly up-regulated and down-regulated cytokine genes for which TMGEAs were available at the time of this analysis.

**Figure 1:**
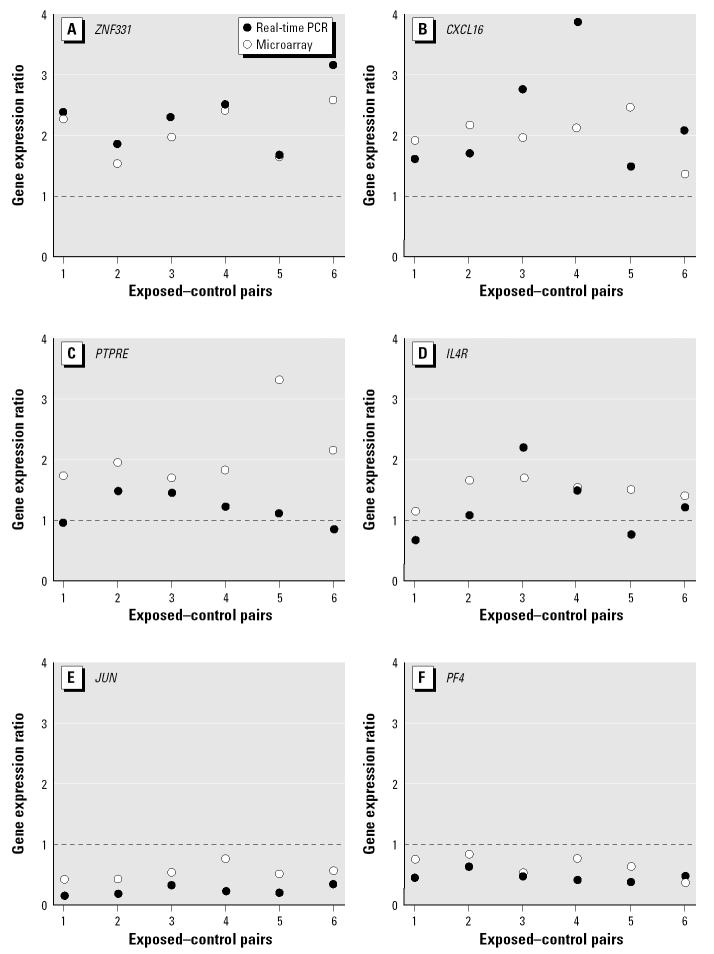
Comparison of GeneChip microarray and real-time PCR data in the six matched exposed–control pairs for the up-regulated genes (*A*) ZNF331, (*B*) *CXCL16*, (*C*) *IL4R*, and (*D*) *PTPRE* and the down-regulated genes (*E*) *JUN* and (*F*) *PF4*: ratios of gene expression in exposed versus control pairs as measured by real-time PCR and microarray.

Real-time PCR of RNA from the six exposed–control pairs tested by GeneChips confirmed that *CXCL16* and *ZNF331* were consistently up-regulated in exposed individuals (mean increases of 103% and 113%, respectively) and that *JUN* and *PF4* were consistently down-regulated in exposed individuals (mean decreases of 81% and 58%, respectively) when compared with unexposed individuals (Figure 1). These differences in expression are very similar to those found by GeneChip analysis (Table 1). Results for *IL4R* and *PTPRE* were less concordant, with increases in some pairs and decreases in others (Figure 1).

### Effect of benzene exposure on the expression of the differentially expressed genes.

Having shown 100% concordance for *CXCL16*, *ZNF331*, *JUN*, and *PF4* between array and real-time data in six matched pairs of benzene-exposed workers and controls, we studied their expression using real-time PCR in a larger set of exposed workers and matched controls (Table 4). RNA from the PBMCs of 13 highly exposed subjects (mean benzene = 43.7 ± 23.9 ppm) and 15 controls was examined (the original six matched pairs of subjects included). The exposed and unexposed subjects were matched on the basis of gender (*p* = 0.7), age (*p* = 0.48), current smoking status, and recent infections (Table 4). We also tested the effect of each covariate, and none negatively confounded (i.e., weakened) the impact of benzene exposure on any of the end points. In this larger data set, *CXCL16* and *ZNF331* were again shown to be very significantly up-regulated, and *JUN* and *PF4* significantly down-regulated (Table 4). Thus, *CXCL16*, *ZNF331*, *JUN*, and *PF4* are four genes clearly identified as being differentially expressed after benzene exposure.

### Lack of potential confounding by changes in lymphocyte subsets.

It is well established that benzene lowers peripheral blood lymphocyte counts (Qu et al. 2002; Rothman et al. 1996), and certain lymphocyte subsets may be more sensitive to benzene’s effects than are others. This raises a concern that our findings could be explained in part by a different distribution of lymphocyte subset populations in workers exposed to benzene compared with controls. To address this potential confounding, we first evaluated the distribution of all measured cell populations that comprise the PBMCs from which mRNA was isolated (Table 5). Total mononuclear cells (i.e., monocytes, CD4^+^ T, CD8^+^ T, CD19^+^ B), lymphocytes, and CD56 (NK) cells were significantly decreased in exposed workers compared with controls (*p* = 0.0052; Table 5). Further, the percentage of total mononuclear cells composed of B cells (i.e., B-cell mononuclear percentage) in the exposed workers was significantly less than that in controls (*p* = 0.0061), whereas the CD8^+^ T-cell mononuclear percentage was significantly increased (*p* = 0.0096). Using linear regression, we determined that the proportion of the mononuclear cell fraction made up by each of the five cell types had no impact on expression of *CXCL16*, *ZNF331*, *JUN*, and *PF4*. Further, the strength and direction of the association between benzene exposure and gene expression were only minimally changed after adjusting for both CD8^+^ T-cell and CD19^+^ B-cell mononuclear cell number and percentages.

## Discussion

To our knowledge, this is the first molecular epidemiologic study to use whole-genome Affymetrix GeneChips for *in vivo* studies of the effects of a specific chemical exposure in humans. A limited number of earlier studies have looked at selected subsets of genes (Wu et al. 2003) or at the effects of smoking (Lampe et al. 2004), but none has examined differences in expression in the transcriptome in the context of benzene exposure. Using a relatively small sample size of six matched pairs of exposed and control subjects, we have been able to identify differentially expressed genes in the PBMC of benzene-exposed individuals that could be confirmed and measured by real-time PCR.

A global analysis of 45,000 probe sets, representing approximately 33,000 well-substantiated human genes, was performed on the GeneChips using stabilized PBMC RNA collected in the field in China as part of a large molecular epidemiology study of benzene-exposed workers (Vermeulen et al. 2004). Although the results will differ based on both the type of processing (e.g., RMA) and adjustment for multiple testing (e.g., FWER with bootstrapping), our results showed that, in the six pairs examined, a potentially large number (> 2,100) of probe sets were (statistically) differentially expressed in the benzene-exposed subjects compared with the control, unexposed subjects. Because the accuracy of this bootstrapping technique is based on asymptotic theory, a 5% FWER is not guaranteed, and thus the statistical results should not be the only criterion for identifying genes for more detailed study. However, by ranking the differentially expressed probe sets identified in the global gene analysis by unadjusted *p*-value, we were able to identify the top 50 that were highly likely to be differentially expressed. We chose four of the known genes from this list for confirmation by real-time PCR. We also increased our probability of finding genes altered by benzene exposure by performing an analysis of the limited subset of 508 cytokine probe sets on the GeneChips. The equivalent analysis of this subgroup indicated that the expression of 19 cytokine genes was significantly different between the exposed and control subjects, and two of these were chosen for confirmation by real-time PCR.

Genes were chosen for real-time PCR confirmation based on the availability of TMGEA, fold change of expression, and expected copy number. Genes thought to be expressed at very low levels were avoided because these have much higher relative measurement error rates (Novak et al. 2002) and because availability of RNA for confirmation was limited. The six genes chosen for further study were *CXCL16*, *JUN*, *PTPRE*, *ZNF331*, *IL4R*, and *PF4*. *TBP* was chosen as the endogenous control gene because recent research suggests it is well suited for real-time PCR investigations of lymphocytes (Lossos et al. 2003) and it is on a different chromosome (6q) from the other genes investigated. This means that gross genetic events (chromosome duplications, deletions, etc.), which may alter the copy number of the genes investigated, will not affect the control gene.

GeneChip findings in the six pairs of subjects for *ZNF331*, *CXCL16*, *PF4*, and *JUN* were all shown to be concordant with real-time PCR data. For *IL4R* and *PTPRE* there was less consistency between the GeneChip findings and those by real-time PCR in the six pairs (Figure 1). Low copy number can be ruled out as an explanation for the discrepancies between GeneChip and real-time PCR findings for *IL4R* and *PTPRE* because they were detected at levels similar to those of the other four genes. One possible explanation is that the Affymetrix probes for these genes are at the 3′ end of transcripts whereas the probes for the *IL4R* and *PTPRE* TMGEAs span exons farther upstream. The different target sequences might explain the discrepancies found in relative expression. Results of real-time PCR using the second-round cRNA used for hybridization to the GeneChips were not significantly different from those described in Figure 1 (data not shown). This suggests that differences were not caused by use of the Small Sample Target Labeling Assay Version II protocol.

In a larger set of 28 study subjects, all four concordant genes were shown to be consistently altered by benzene exposure: *CXCL16* and *ZNF331* were up-regulated, whereas *JUN* and *PF4* were down-regulated. Alteration in the expression of any of the four genes could be a consequence of upstream events. However, it is also possible that germline variation in one or more regulatory regions of these four genes could be particularly susceptible to the effects of benzene exposure. Further studies are needed to investigate genetic variation across each of these genes to determine if one or more variants could be functionally important in benzene exposure. The identification of interactions between genetic variants and benzene’s effects could lead to further insights into the mechanisms associated with benzene-induced leukemia and other hematologic diseases.

*CXCL16* is also known as *SR-PSOX* or *CXCLG16* and maps to chromosome 17p13. It encodes chemokine (C-X-C motif) ligand 16, a scavenger receptor that mediates adhesion and phagocytosis of both Gram-negative and Gram-positive bacteria. This facilitates the uptake of various pathogens and chemotaxis of T cell and NK T cells by antigen-presenting cells through its chemokine domain (Shimaoka et al. 2003). ZNF331 is a member of the Krüppel-related family of zinc finger proteins that contain a Krüppel associated box (KRAB) domain and is likely a transcriptional repressor (Meiboom et al. 2003). The *ZNF331* gene lies close to a frequent breakpoint region of follicular thyroid adenomas (Meiboom et al. 2003), but the question of why benzene should so markedly affect *ZNF331* expression remains unclear at present.

The JUN, FOS, MAF, and A TF subfamilies are dimeric, basic region–leucine zipper proteins that make up the AP-1 transcription factor. AP-1 transcription factors (Shaulian and Karin 2002) are involved in both the induction and prevention of apoptosis, depending on tissue type (Shaulian and Karin 2002). As part of AP-1, JUN is primarily a positive regulator of proliferation. JUN-deficient fibroblasts have marked proliferative defects *in vitro* (Schreiber et al. 1999; Wisdom et al. 1999), and proliferation of JUN-deficient hepatocytes is severely impaired during liver regeneration *in vivo* (Bakiri et al. 2000; Behrens et al. 2002; Schreiber et al. 1999). In mouse erythroleukemia and fibroblast cells, inhibition of *fos* and *jun* has demonstrated their requirement for proliferation and cell-cycle progression (Shaulian and Karin 2001). The lower levels of JUN could thus be indicative that the PBMCs of benzene-exposed individuals are not proliferating or progressing through the cell cycle as quickly as those of nonexposed individuals.

PF4, also known as CXCL4, is a polypeptide constituent of platelet alpha granules that is released during platelet aggregation and inhibits heparin-mediated reactions. PF4 has been shown to have numerous other biologic properties, including inhibiting endothelial cell proliferation, migration, and angiogenesis (Gupta and Singh 1994; Maione et al. 1990; Niewiarowski et al. 1976) and inhibiting T-cell function by down-modulating cell proliferation and cytokine release (Fleischer et al. 2002). PF4 is expressed exclusively in platelets, megakaryocytes, and their precursors (Doi et al. 1987), and its down-regulation in benzene-exposed workers probably reflects decreased expression in platelets or progenitor cells because they are present in the PBMC fraction.

We explored whether increased and decreased expression of these genes after benzene exposure was a reflection an alteration in the subset make up of the PBMC population. Although benzene exposure did cause changes in the subset makeup of the PBMC fraction (Table 5), these changed proportions had no impact on expression of *CXCL16*, *ZNF331*, *JUN*, and *PF4*, and the strength and direction of the association between benzene exposure and gene expression was minimally changed after adjusting for both CD8^+^ T-cell and B-cell mononuclear cell counts and percentages. Thus, the altered expression was not likely to be caused by changes in the make up of the PBMC fraction. Unfortunately, the subjects studied here were selected to have a high level of benzene exposure to make this initial exploratory effort as efficient as possible by maximizing the contrast between the exposed workers and controls. Consequently, the exposure range was too narrow to be able to detect a dose–response relationship among exposed workers, which was not a goal of this study. In the future, we plan to analyze substantially more samples selected from workers with a wide range of benzene exposure to allow us to construct a detailed model of the dose–response relationship.

Generating relative expression using RMA combined with a bootstrapping method for controlling the FWER appears to be an effective way to identify genes associated with chemical exposure. The relative expression of a subset of six genes (all selected as statistically differentially expressed from GeneChips) were confirmed by real-time PCR in either all or most of the six exposed–unexposed pairs analyzed and in a larger data set from 28 subjects. There was also remarkable consistency between the real-time data and the differential expression ratios calculated by RMA for at least four of these genes. Larger data sets will be needed if we are to characterize a pattern of gene expression related to benzene exposure using machine-learning algorithms. However, we did attempt to explore gene ontology with the program EASE (Expression Analysis Systematic Explorer; Hosack et al. 2003) using the current data set and found that immune response genes gave the largest number of significant population hits, supporting our decision to analyze cytokine genes as a subset.

In conclusion, we have shown that microarray analysis can be a good tool for discovering genes of potential mechanistic interest or biomarkers of exposure and early effect in molecular epidemiologic studies of populations exposed to potential carcinogens. Further, only small numbers of paired study subjects are required to identify differentially expressed genes, making such studies cost-effective. The small-sample protocol used here also limits the amount of high-quality RNA required, meaning that archived samples, stored by partial isolation and stabilization of the RNA in the field, are amenable to analysis. These studies therefore provide a model for biomarker discovery in chemically exposed human populations, although with lower exposed populations it may be necessary to study more subject pairs, with 15 pairs probably being ideal. Because the price of global gene expression arrays is decreasing, such studies are becoming more feasible.

## Figures and Tables

**Table 1:** List of top 25 probe sets up-regulated by benzene exposure identified on U133 chips.*^a^*

Probe set ID	Gene symbol	Location	Gene description	*p*-Value	Ratio (GM)
207630_s_at	*CREM*	10p12.1–p11.1	cAMP responsive element modulator	3.97 × 10^−4^	2.45
221840_at	*PTPRE*	10q26	protein tyrosine phosphatase receptor type E*^b^*	7.77 × 10^−4^	2.07
219228_at	*ZNF331*	19q13.3–q13.4	zinc finger protein 331*^b^*	4.49 × 10^−4^	2.02
204924_at	*TLR2*	4q32	toll-like receptor 2	5.09 × 10^−4^	2.01
227613_at	*ZNF331*		zinc finger protein 331	3.73 × 10^−4^	1.97
223454_at	*CXCL16*	17p13	chemokine (C-X-C motif) ligand 16*^b^*	3.93 × 10^−4^	1.96
228962_at	*PDE4D*		phosphodiesterase 4D, cAMP-specific (phosphodiesterase E3 dunce homolog, Drosophila)	7.11 × 10^−4^	1.85
214696_at	*MGC14376*	17p13.3	hypothetical protein MGC14376	1.71 × 10^−4^	1.78
210732_s_at	*LGALS8*	1q42–q43	lectin, galactoside-binding, soluble, 8 (galectin 8)	5.49 × 10^−4^	1.76
212371_at	*PNAS-4*		CGI-146 protein	7.61 × 10^−4^	1.7
225390_s_at	*KLF13*		Krüppel-like factor 13	4.34 × 10^−4^	1.69
227645_at	*P101*-*PI3K*	17p13.1	phosphoinositide-3-kinase, regulatory subunit, polypeptide p101	4.71 × 10^−4^	1.66
226652_at	*USP3*		ubiquitin specific protease 3	5.48 × 10^−4^	1.64
221641_s_at	*ACATE2*	Xp22.13	likely ortholog of mouse acyl-coenzyme A thioesterase 2, mitochondrial	6.01 × 10^−4^	1.63
202055_at	*KPNA1*		karyopherin alpha 1 (importin alpha 5)	2.89 × 10^−4^	1.61
226743_at	*FLJ34922*	17q12	hypothetical protein FLJ34922	7.42 × 10^−4^	1.6
228393_s_at	*ZNF302*		zinc finger protein 302	6.64 × 10^−4^	1.58
225120_AT	*PURB*		purine-rich element binding protein B	3.90 × 10^−4^	1.58
218515_at	*C21orf66*	21q21.3	chromosome 21 open reading frame 66	5.80 × 10^−4^	1.56
202224_at	*CRK*		v-crk sarcoma virus CT10 oncogene homolog (avian)	6.45 × 10^−5^	1.55
200614_at	*CLTC*	17q11–qter	clathrin, heavy polypeptide (Hc)	6.33 × 10^−4^	1.55
212014_x_at	*CD44*	11p13	CD44 antigen (homing function and Indian blood group system)	3.08 × 10^−4^	1.54
223461_at	*TBC1D7*	6p23	TBC1 domain family, member 7	6.75 × 10^−4^	1.51
209835_x_at	*CD44*	11p13	CD44 antigen (homing function and Indian blood group system)	2.69 × 10^−4^	1.51
213315_x_at	*LOC91966*	Xq28	hypothetical protein LOC91966	7.54 × 10^−4^	1.49

GM, geometric mean.

a Top 25 up-regulated probe sets were selected by RMA on the basis of *p*-value and then ranked according to expression ratio. Gene annotations are from NetAffx (http://www.affymetrix.com/analysis/index.affx).

b Genes chosen for further analysis by real-time PCR.

**Table 2:** List of top 25 probe sets down-regulated by benzene exposure identified on U133 chips.*^a^*

Probe set ID	Gene symbol	Location	Gene description	*p*-Value	Ratio (GM)
200800_s_at	*HSPA1A*	6p21.3	heat shock 70 kDa protein 1A	4.38 × 10^−4^	0.34
242904_x_at	*MGC8721*	8p12	hypothetical protein MGC8721	4.42 × 10^−4^	0.43
213281_at	*JUN*	1p32–p31	v-jun sarcoma virus 17 oncogene homolog (avian)*^b^*	6.56 × 10^−4^	0.51
229264_at	*FLJ39739*		FLJ39739 protein (M. musculus) S00030 neurofilament triplet M protein, mouse	3.06 × 10^−4^	0.56
237510_at	*MYNN*	3q26.31	myoneurin	2.17 × 10^−4^	0.59
229054_at	*FLJ39739*		FLJ39739 protein	6.64 × 10^−4^	0.59
202732_at	*PKIG*	20q12–q13.1	protein kinase (cAMP-dependent, catalytic) inhibitor gamma	3.24 × 10^−4^	0.65
229872_s_at	*KIAA0493*	8q24.13	Homo sapiens cDNA FLJ39739 fis, clone SMINT2016440	1.48 × 10^−4^	0.67
230574_at			Homo sapiens transcribed sequences	1.13 × 10^−4^	0.7
224495_at	*MGC10744*	17p13.1	hypothetical protein MGC10744	8.17 × 10^−4^	0.73
243_g_at	*MAP4*	3p21	microtubule-associated protein 4	1.41 × 10^−4^	0.75
244741_s_at	*MGC9913*	19q13.43	hypothetical protein MGC9913	4.67 × 10^−4^	0.76
221419_s_at				6.82 × 10^−4^	0.77
219503_s_at	*FLJ11036*	3p25.1	hypothetical protein FLJ11036	1.21 × 10^−4^	0.77
240406_at	*USP16*	21q22.11	ubiquitin specific protease 16	4.19 × 10^−4^	0.8
241749_at		9q31.1	similar to RIKEN cDNA 2310039E09	2.96 × 10^−4^	0.81
228932_at			Homo sapiens transcribed sequence with moderate similarity to protein sp:P39194 (H. sapiens) ALU7_HUMAN Alu subfamily SQ sequence contamination warning entry	4.08 × 10^−4^	0.82
227667_at			Homo sapiens transcribed sequence with weak similarity to protein pir:B36298 (H. sapiens) B36298 proline-rich protein PRB3S (cys)—human (fragment)	1.56 × 10^−4^	0.82
239063_at			Homo sapiens cDNA FLJ39803 fis, clone SPLEN2007794	3.51 × 10^−5^	0.83
236509_at			Homo sapiens transcribed sequences	4.61 × 10^−4^	0.83
200655_s_at	*CALM1*	14q24–q31	calmodulin 1 (phosphorylase kinase, delta)	1.23 × 10^−4^	0.83
221384_at	*UCP1*	4q28–q31	uncoupling protein 1 (mitochondrial, proton carrier)	9.86E-06	0.84
203834_s_at	*TGOLN2*	2p11.2	trans-Golgi network protein 2	5.44 × 10^−4^	0.85
225122_at	*RNF31*	14q11.2	ring finger protein 31	1.87 × 10^−4^	0.86
229975_at			Homo sapiens transcribed sequence with weak similarity to protein ref:NP_060312.1	8.19 × 10^−4^	0.86

GM, geometric mean.

a Top 25 down-regulated probe sets were selected by RMA on the basis of *p*-value and then ranked in the table according to expression ratio. Gene annotations are from NetAffx (http://www.affymetrix.com/analysis/index.affx).

b *JUN* was chosen for further analysis by real-time PCR.

**Table 3:** List of cytokine probe sets identified by U133 GeneChips from the cytokine subset that were significantly different in benzene-exposed and unexposed individuals.*^a^*

Probe set ID	Gene symbol	Gene description	*p*-Value	Ratio (GM)
243296_at	*PBEF*	pre-B-cell colony-enhancing factor	0.0275	2.17
211676_s_at	*IFNGR1*	interferon gamma receptor 1	0.0037	2.16
223454_at	*CXCL16*	chemokine (C-X-C motif) ligand 16*^b^*	0.0004	1.96
217738_at	*PBEF*	pre-B-cell colony-enhancing factor	0.0206	1.93
201408_at	*PPP1CB*	protein phosphatase 1, catalytic subunit, beta isoform	0.0017	1.72
213743_at	*CCNT2*	cyclin T2	0.0080	1.68
226333_at	*IL6R*	interleukin 6 receptor	0.0042	1.57
209827_s_at	*IL16*	interleukin 16 (lymphocyte chemoattractant factor)	0.0025	1.53
203233_at	*IL4R*	interleukin 4 receptor	0.0009	1.49
228222_at	*PPP1CB*	protein phosphatase 1, catalytic subunit, beta isoform	0.0206	1.43
205945_at	*IL6R*	interleukin 6 receptor	0.0269	1.4
204912_at	*IL10RA*	interleukin 10 receptor, alpha	0.0044	1.39
205291_at	*IL2RB*	interleukin 2 receptor, beta	0.0113	1.39
224914_s_at	*CIP29*	cytokine induced protein 29 kDa	0.0115	1.38
202727_s_at	*IFNGR1*	interferon gamma receptor 1	0.0146	1.32
204773_at	*IL11RA*	interleukin 11 receptor, alpha	0.0111	1.23
204645_at	*CCNT2*	cyclin T2	0.0149	1.22
223961_s_at	*CISH*	cytokine inducible SH2-containing protein	0.0244	1.16
206359_at	*SOCS3*	suppressor of cytokine signaling 3	0.0229	1.08
Down-regulated gene expression				
210354_at	*IFNG*	interferon, gamma	0.0149	0.35
206390_x_at	*PF4*	platelet factor 4 [chemokine (C-X-C motif) ligand 4]*^b^*	0.0108	0.62
209767_s_at	*GP1BB*	glycoprotein Ib (platelet), beta polypeptide	0.0204	0.77
201896_s_at	*DDA3*	differential display and activated by p53	0.0232	0.8
242254_at		Homo sapiens transcribed sequence with moderate similarity to protein ref:NP_071431.1 (H. sapiens) cytokine receptor-like factor 2; cytokine receptor CRL2 precursor	0.0187	0.84
213258_at	*TFPI*	tissue factor pathway inhibitor (lipoprotein-associated coagulation inhibitor)	0.0124	0.85
244848_at		CDNA FLJ31075 fis, clone HSYRA2001484	0.0282	0.88
235889_at		Homo sapiens transcribed sequence with moderate similarity to protein ref:NP_060312.1 (H. sapiens) hypothetical protein FLJ20489	0.0131	0.89
243438_at	*PDE7B*	phosphodiesterase 7B	0.0019	0.91

GM, geometric mean.

a Gene annotations are from NetAffx (http://www.affymetrix.com/analysis/index.affx).

b Gene chosen for further analysis by real-time PCR.

**Table 4:** Gene expression measured by real-time PCR in the larger set of 28 benzene-exposed workers and controls.

Characteristics	Control (*n* = 15)	Exposed (*n* = 13)	*p*-Value*^a^*	*p*-Value*^b^* (Unadjusted)
Sex				
Male	8 (53%)	6 (46%)		
Female	7 (47%)	7 (54%)		
Age (years)				
Mean ± SD	32.5 ± 8.8	35.1 ± 6.1		
Median	33	36		
Current smoking				
Yes	5 (33%)	4 (31%)		
No	10 (67%)	9 (69%)		
Recent infection				
Yes	1 (7%)	1 (8%)		
No	14 (93%)	12 (92%)		
Benzene exposure				
Air level (ppm)	< 0.04	43.66 ± 23.87		
Urine level (μg/L)	0.36 ± 0.51	778.70 ± 1433.02		
Up-regulated genes				
CXCL16	2.37 ± 0.62*^c^*	3.66 ± 0.54	0.00001	< 0.0001
	2.55*^d^*	3.9		
ZNF331	1.50 ± 0.70	3.00 ± 0.91	0.00001	< 0.0001
	1.61	2.92		
Down-regulated genes				
JUN	6.29 ± 1.00	4.94 ± 1.26	0.0037	0.0038
	6.57	4.66		
PF4	6.05 ± 0.90	5.46 ± 1.35	0.052	0.012*^e^*
	6.24	5.18		

a By Wilcoxon exact test.

b Analyzed by linear regression. Results are unadjusted because age, sex, current smoking, recent infections, and alcohol use did not weaken the effect of benzene exposure on any gene transcript.

c Data presented as mean ± SD.

d Data presented as median.

e One subject with an extreme outlier value was excluded from the regression analysis.

**Table 5:** Subsets of lymphocytes and monocytes in the PBMC fraction of the 28 subjects comprising the larger study population.

Cell types	Control (*n* = 15)	Exposed (*n* = 13)	*p*-Value*^a^*
Mononucleocytes/L	2240 ± 540*^b^*	1704 ± 393	0.0052
Monocytes/mononucleocytes (%)	8.24 ± 2.43	8.53 ± 2.06	0.56
CD4^+^ T cells/mononucleocytes (%)	34.08 ± 7.95	33.01 ± 5.62	0.86
CD8^+^ T cells/mononucleocytes (%)	25.32 ± 6.98	33.15 ± 7.13	0.0096
CD19 cells/mononucleocytes (%)	10.14 ± 2.27	6.79 ± 5.00	0.0061
CD56 cells/mononucleocytes (%)	22.22 ± 10.42	18.52 ± 4.20	0.62

a By Wilcoxon exact test.

b Data presented as mean ± SD.
